# An unusual presentation of LCAT deficiency as nephrotic syndrome with normal serum HDL-C level

**Published:** 2016-01-05

**Authors:** Manish R Balwani, Vijaykumar A Ghodela, Vivek B Kute, Pankaj R Shah, Himanshu V Patel, Dinesh N Gera, Aruna Vanikar, Hargovind L Trivedi

**Affiliations:** ^1^Department of Nephrology and Clinical Transplantation, Laboratory Medicine, Transfusion Services and Immunohematology, Institute of Kidney Diseases and Research Center, Dr. HL Trivedi Institute of Transplantation Sciences (IKDRC-ITS), Ahmedabad, India; ^2^Department of Pathology, Laboratory Medicine, Transfusion Services and Immunohematology, Institute of Kidney Diseases and Research Center, Dr. HL Trivedi Institute of Transplantation Sciences, Ahmedabad, India

**Keywords:** LCAT deficiency, Nephrotic syndrome, Corneal deposit, Anemia

## Abstract

Clinical and biochemical manifestations of lecithin-cholesterol acyltransferase (LCAT) deficiency include an abnormal lipid profile (characterized by hypercholesterolemia with markedly decreased high-density lipoprotein cholesterol [HDL-C] and hypertriglyceridemia), corneal opacities, hematologic abnormalities (normochromic anemia of varying intensity), splenomegaly, variable early coronary artery disease and nephropathy (initially proteinuria followed by progressive deterioration of renal function). We presented a patient with nephrotic syndrome, which renal biopsy revealed classic features of LCAT deficiency. To our knowledge, the present case is the first reported case of LCAT deficiency presenting with symptoms related to nephrotic syndrome in a patient with no obvious family history without any corneal deposits and normal HDL-C levels.

**Implication for health policy/practice/research/medical education:**

Lecithin-cholesterol acyltransferase (LCAT) deficient patients may present with renal disease only even with normal high-density lipoprotein cholesterol (HDL-C) levels. Hence, it needs high index of suspicion for LCAT deficiency when renal biopsy is suggestive of lamellar foamy deposits in mesangium and capillary walls even if serum HDL-C levels are normal.

## Introduction

Lecithin-cholesterol acyltransferase (LCAT) is a plasma enzyme that transesterifies cholesterol and phosphatidylcholines (lecithins) into cholesteryl esters and lysophosphatidylcholine (1). LCAT acts on both low-density lipoproteins (apolipoprotein [apo] B–containing lipoproteins like low-density lipoprotein [LDL], very low-density lipoprotein [VLDL]) and high-density lipoproteins (HDLs), corresponding to the β- and α-activity of LCAT, respectively (2). LCAT binds to HDL particles preferentially which contain apoA-I, which is the main activator of enzyme, and is responsible for most of the cholesteryl ester synthesis (2,3). HDL promotes the efflux of excess cholesterol from peripheral tissues and for biliary excretion, returns it to the liver (4). Nascent HDL is secreted by liver and intestine as lipid-poor apoA-I and undergoes dynamic remodeling and intravascular maturation. LCAT plays a key role in this maturation process. The LCAT deficiency can be either acquired, usually secondary to liver disorder, or congenital with an autosomal recessive mode of inheritance. Mutations in the LCAT gene result in either milder phenotype known as fish-eye disease or severe familial LCAT deficiency (5). Mutated LCAT genotype results in a gene-dose–dependent alteration in plasma lipid/lipoprotein profile, hematologic abnormalities and renal disease with or without premature cardiovascular disease (6-8). Patients with familial LCAT deficiency have complete loss of LCAT activity with an increased proportion of unesterified cholesterol in plasma with markedly reduced HDL-C. Clinical features include renal disease with proteinuria, with progression to end-stage renal disease (ESRD), corneal opacification and anemia (5). In fish-eye disease, there is a partial loss of LCAT enzyme activity, a selective loss of HDL associated α-LCAT activity and preserved activity toward apolipoprotein B-containing lipoprotein. It causes normal to slightly elevated free cholesterol in plasma with marked reduction in HDL cholesterol and corneal opacification without renal disease (9,10). Premature coronary artery disease is not seen in most familial LCAT deficiency cases but may be present in few of patients with familial LCAT deficiency (8). Patients with LCAT deficiency show inhomogeneous tissue and plasma lipoprotein abnormalities (11). Lesions are found in commonly affected tissues like kidney, spleen, cornea, and erythrocytes, probably due to lipid abnormalities (12,13). Kidney disease is a major cause of morbidity and mortality, where ESRD is a common outcome (14,15). Ultrastructural analysis of kidney shows expansion of mesangium and peripheral basement membranes with irregular vacuoles containing highly osmiophilic “lamellar bodies” (16). Altered phospholipid composition of erythrocytes may lead to mild anemia due to phagocytosis (17). We report a patient who presented with symptoms related to nephrotic syndrome and had an abnormal lipid profile with normal HDL-C and without corneal deposits who was finally diagnosed with LCAT deficiency. This case describes the spectrum of biochemical and renal manifestations of LCAT deficiency with associated histologic and ultrastructural findings.

## Case Presentation

**Figure 1:**
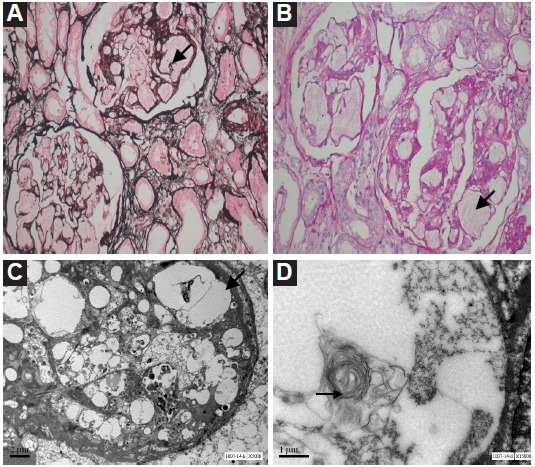
Histologic findings on renal biopsy. (A-B) Light microscopy findings. Glomeruli show markedly dilated capillary lumina filled with lamellated fluid (arrows), (A: Jone’s silver-methenamine staining, B: periodic acid–Schiff staining). (C-D) Electron microscopy findings. Lipid deposits are observed in the mesangial area and lamina densa of basement membranes (C). Cystically dilated capillary loop showing concentrically lamellated/myelinoid structure. Original magnification ×200 in A, ×200 in B, ×5000 in C and ×15000 in D‏.

A 28-year-old female was admitted to our hospital with history of gradually progressive pedal edema and weight gain of 4 kg since 4 months. There was no other complaint on presentation. The family history was insignificant. On physical examination, she had normal vitals along with normal systemic examination except bilateral pitting pedal edema. Ophthalmic examination including fundoscopy was unremarkable. Laboratory data on admission showed hemoglobin; 9.3 g/dl, mean corpuscular volume (MCV); 82.6 fl, white blood count (WBC); 9690/µl with a normal differential count. Additionally poikilocytosis and ‘target’ erythrocytes in peripheral blood smear was seen. Around 3.3 g/day proteinuria was also found, while hematuria was not existed. Serum blood urea nitrogen and creatinine were 24 mg/dl and 0.99 mg/dl respectively. Serum total proteins were 4.5 g/dl (serum albumin 2.5 g/dl). Liver function tests and lactate dehydrogenase levels, prothrombin time, and partial thromboplastin time were within normal limits. She had a raised total serum cholesterol (415 mg/dl) and triglycerides (402 mg/dl) levels. There was also high serum VLDL (80.40 mg/dl) and normal HDL-C (63 mg/dl). Coombs test and autoantibodies was negative. Serum complements consisting C3 and C4, was within the normal range. A renal biopsy was conducted to find the etiology of nephrotic syndrome. Light microscopy showed 22 glomeruli with variable affection and enlarged size (Figure 1). Normal glomerular architecture was replaced by markedly dilated capillary lumina, which was filled with a lamellated, pink staining, fluid-like material. Mesangial matrix was focally increased and showed bubbly appearance. Capillary membranes showed craters on silver staining. Tubules showed moderate degeneration. Electron microscopy examination showed lipid deposition in the lamina densa of the basement membranes. The cystically dilated capillary loops revealed concentrically lamellated/myelinoid structures. Glomeruli were enlarged due to marked accumulation of amorphous lipid material in the capillary loops. Immunofluorescence examination of renal biopsy specimen was not done due to inadequate sample size. Finally, the evaluation of the plasma LCAT enzymatic activity revealed markedly decreased LCAT activity (10.1 nmol/ml/h; reference range, 80.9 ± 11.2 nmol/ml/h). We confirmed again the absence of corneal opacities or any kind of ophthalmic involvement. The patient was treated with a combination of gemfibrozil, atorvastatin, and telmisartan without any significant improvement in proteinuria at 2 months of follow-up.

## Discussion

Familial LCAT deficiency was initially described in people of northern European origin and subsequently in patients from different geographic areas, including Japan and North America (7,8,18,19). Variable clinical and biochemical manifestations of LCAT deficiency include an abnormal lipid profile (characterized by hypercholesterolemia with markedly decreased HDL-C and hypertriglyceridemia), corneal opacities, hematologic abnormalities (normochromic anemia of varying intensity), splenomegaly, variable early coronary artery disease and nephropathy (initially proteinuria followed by progressive deterioration of renal function) (8,17,20). In the current case, we made a working diagnosis of nephrotic syndrome with secondary hyperlipidemia. The diagnosis of LCAT deficiency was made by retrospective evaluation after renal biopsy findings which were typical of LCAT deficiency. She was subsequently found to have an abnormal lipid profile characterized by high total cholesterol, high triglycerides, low to normal HDL-C, and low esterified cholesterol. The low HDL-cholesterol level is thought to be responsible for the less efficient reverse cholesterol transport and so accumulation of cholesterol in tissue (21). Along with removal of cholesterol from peripheral tissue, HDL-C also removes oxidized lipids from peripheral tissue (22). Consequently, oxidized lipids and lipid aggregates cause the activation of macrophages and thus promoting foamy histiocytes formation (23). Mild anemia with target cells was seen in our patient. Anemia is probably due to slight hemolysis in conjunction with insufficient erythropoiesis (18). The erythrocytes of patients with LCAT deficiency have structural and functional abnormalities, like decreased osmotic fragility and altered phospholipid composition (24). These abnormalities in lipid composition may lead to phagocytosis and thus mild anemia (17). Renal disease is a major cause of morbidity and mortality in patients with LCAT deficiency. Nephrotic syndrome frequently progresses to ESRD necessitating dialysis and kidney transplantation (14,15). Our patient was found to have the typical early renal manifestations and corresponding pathologic findings that are related to the abnormal lipid metabolism in patients with LCAT deficiency. Abnormal storage of lipids in kidney may occur in a number of disorders either due to an inborn error of metabolism or as a consequence of metabolic alteration such as in nephrotic syndrome (25). The characteristic light microscopic findings (i.e., mesangial expansion, capillary wall thickening, and vacuolation) and the ultrastructural appearance (lipid deposits in many areas including subendothelium and mesangium, and also lamellar structures) of the kidney specimen from our patient are typical findings in LCAT deficiency (25). Clinical features of patients with LCAT deficiency may vary even among the members of the same family (26). We have treated our patient with lipid lowering agents and angiotensin converting enzyme inhibitors, however, there has been no improvement after two months of follow-up. There is no specific treatment established yet for familial LCAT deficiency. Transplantation of the cornea or kidney was performed in cases with severe disease, although the disease has been reported to recur in the transplanted kidney (27). To our knowledge, the present case is the first reported case of LCAT deficiency presenting with symptoms related to nephrotic syndrome in a patient with no obvious family history without any corneal deposits and normal HDL-C levels. We could not find the reason for normal HDL-C in this patient. This case describes a spectrum of biochemical and renal manifestations of LCAT deficiency with histological and ultrastructural correlation. LCAT deficient patients may present with renal disease even with normal HDL-C levels. Hence, it needs high index of suspicion for LCAT deficiency when renal biopsy is suggestive of lamellar foamy deposits in the capillary lumina, mesangium and capillary walls, even if serum HDL-C levels are normal.

## Conclusion

LCAT deficiency usually presents as renal disease with proteinuria, ESRD, corneal opacification, hypertriglyceridemia, low HDLC levels and anemia. In our case, LCAT deficient patient presented with nephrotic syndrome with no evidence of corneal deposits and with normal HDL-C levels.

## Authors’ contribution

All authors wrote the paper equally.

## Conflicts of interest

The authors declared no competing interests.

## Ethical considerations

Ethical issues (including plagiarism, data fabrication, double publication) have been completely observed by authors.

## Funding/Support

None.
